# Heterotopic Pancreas Located at the Gastroesophageal Junction in a Hiatal Hernia: A Case Report

**DOI:** 10.7759/cureus.20630

**Published:** 2021-12-23

**Authors:** Joshua K Jenkins, Forest Smith, Stephen Mularz, Shweta Chaudhary

**Affiliations:** 1 General Surgery, Lincoln Memorial University-DeBusk College of Osteopathic Medicine, Harrogate, USA; 2 Internal Medicine, Hazard Appalachian Regional Healthcare (ARH) Regional Medical Center, Hazard, USA; 3 Gastroenterology, Hazard Appalachian Regional Healthcare (ARH) Regional Medical Center, Hazard, USA; 4 Pathology, Hazard Appalachian Regional Healthcare (ARH) Regional Medical Center, Hazard, USA

**Keywords:** gastroesophageal junction, pancreatic rest, hiatal hernia, ectopic pancreas, heterotopic pancreas

## Abstract

Heterotopic pancreas, commonly referred to as pancreatic rest or ectopic pancreas, is a congenital anomaly in which pancreatic tissue is anatomically separate from the main pancreatic gland without continuity of a duct system or vascularity. It is commonly found in the upper gastrointestinal tract, specifically in the stomach and small intestine. To date, only about 18 adult cases of heterotopic pancreas in the esophagus have been reported in the English medical literature; seven of which were in women, and five of which originated at the gastroesophageal junction (GEJ). Of these five cases, only two occurred in a hiatal hernia. We report the third case of the heterotopic pancreas at the GEJ in a hiatal hernia discovered in a 62-year-old Caucasian female who presented to the outpatient clinic complaining of worsening reflux characterized as burning retrosternal chest pain. The patient failed maximum medical therapy and was referred to general surgery for laparoscopic hiatal hernia repair with Toupet fundoplication to prevent further reflux and damage to the esophagus. The reflux symptoms persisted after the procedure. Follow-up esophagogastroduodenoscopy with biopsy of the GEJ revealed a small focus of heterotopic pancreas tissue, confirmed by histopathology. The management of heterotopic pancreas differs throughout the literature depending on the size, symptomatology, and potential for malignancy. Management in cases of pancreatic rest, specifically at the GEJ, ranges from observation with conservative medical therapy, resection, or esophagectomy. With this case, we aim to contribute to the literature with the third case of pancreatic rest in the GEJ of a hiatal hernia.

## Introduction

Heterotopic pancreas, commonly referred to as pancreatic rest or ectopic pancreas, is a congenital anomaly in which pancreatic tissue is anatomically separate from the main pancreatic gland without continuity of a duct system or vascularity. It is most commonly located in the upper gastrointestinal tract, specifically, in the stomach, duodenum, and proximal jejunum. Although it has been observed in other anatomical locations, such as the esophagus, ileum, Meckel's diverticulum, and biliary tree, these cases are very rare. Reports are more common amongst adult males, with incidence peaking during the fourth, fifth, and sixth decades of life [1]. Complications of heterotopic pancreas reported in the upper gastrointestinal system range from inflammation of the ectopic tissue, abscess, endocrine dysfunction, malignant degeneration, mechanical obstruction, and bleeding [2].

The embryologic basis of pathogenesis for heterotopic pancreas is multifaceted with ideologies described by the misplacement theory, metaplasia theory, and totipotent cell theory. According to misplacement theory, fragments become separated from the pancreas during foregut rotation of embryogenesis and then develop into mature elements later in life [3]. The pancreatic rests are most likely to drop off from the dorsal primordium, which is most likely the reason this phenomenon is observed in the stomach, duodenum, and proximal jejunum. This does not, however, explain why these pancreatic rests are observed in other sites [4]. The totipotent cell theory, however, suggests that the endodermal cells lining the gut differentiate into pancreatic tissue themselves [1]. This is a more reliable explanation for the presence of pancreatic rests in the lesser common sites, such as Meckel’s diverticulum, ampulla of Vater, fallopian tube, and mediastinum, since these tissues are derived from the omphalomesenteric duct remnant, where the cells lining this duct are known to be pluripotent, expressing either gastric, pancreatic, hepatic, or other terminal endoderm-derived phenotypes [4]. The metaplasia theory, on the other hand, suggests that endodermal tissues migrate to the submucosa during embryogenesis and transform into pancreatic tissue [1].

We report a rare case of esophageal heterotopic pancreas located at the GEJ of a female patient discovered incidentally on upper endoscopy for worsening gastroesophageal reflux disease (GERD) symptoms. To date, only about 18 adult cases of heterotopic pancreas in the esophagus have been reported in the English medical literature; seven of which were in women, and five of which originated at the GEJ. This work has been reported in line with the Surgical Case Report (SCARE) criteria [5].

## Case presentation

A 62-year-old Caucasian female presented to the outpatient clinic complaining of worsening reflux and burning retrosternal pain. Over-the-counter antacids gave her no relief, and she had started to experience a dry cough and changes in her voice. Her symptoms were exacerbated in the supine position. There was no relation of her symptoms to the consumption of food products, but she did mention sometimes feeling the sensation of food sticking in the middle of her throat during swallowing. She also noted associated bloating, the inability to belch, and infrequent stool habits. Her past medical history was significant for borderline diabetes mellitus, GERD, hiatal hernia, and hyperlipidemia. The patient’s medications at the time included calcium carbonate 500-mg once daily and omega-3 fatty acids (fish oil concentrate) 1,000-mg once daily. The patient’s family history was significant for colorectal carcinoma in her father. She was a former smoker with a stop date greater than 20 years prior and admitted to occasionally consuming alcohol. On physical exam, the patient did not appear to be in any acute distress. She had no tachypnea or diaphoresis at rest. She did not appear pale or jaundiced. Her abdomen was soft and non-tender with no distention or rigidity.

**Figure 1:**
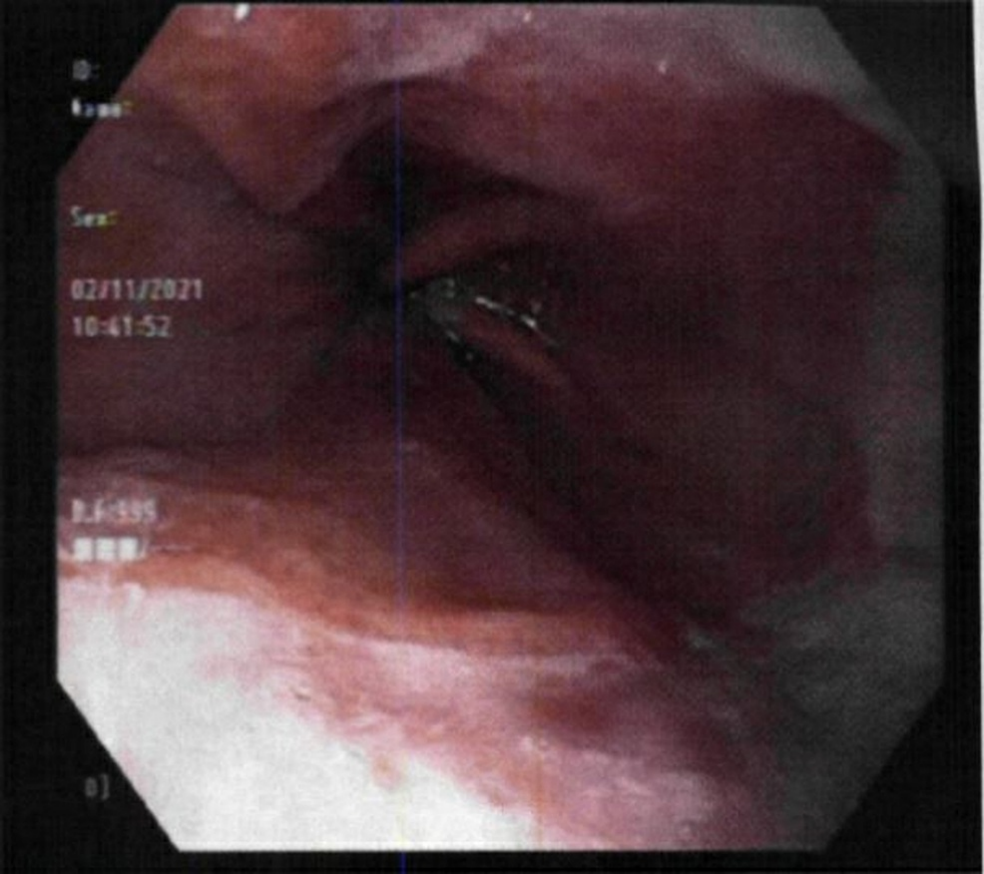
Endoscopic image revealing an irregular gastroesophageal junction with inflammation.

Due to the sudden worsening of her symptoms, the patient was started on medical therapy with famotidine 40-mg at bedtime, pantoprazole 40-mg daily, and a two-week course of sucralfate before meals and at bedtime. Given the severity of her symptoms, there was a concern for severe esophagitis due to reflux or even possible ulcers within the hiatal hernia itself. An esophagogastroduodenoscopy (EGD) was scheduled for further evaluation, which confirmed a diaphragmatic hernia without obstruction and an irregular, inflamed gastroesophageal junction (GEJ) (Figure 1). Biopsy results revealed intestinal metaplasia and focal atypia with indefinite dysplasia in the GEJ and chronic gastritis in the antrum of the stomach. Due to histology noting indefinite dysplasia, the sample was sent to integrated oncology for case consultation. The sample was then confirmed as intestinal metaplasia and focal acute inflammation with no malignant involvement.

**Figure 2:**
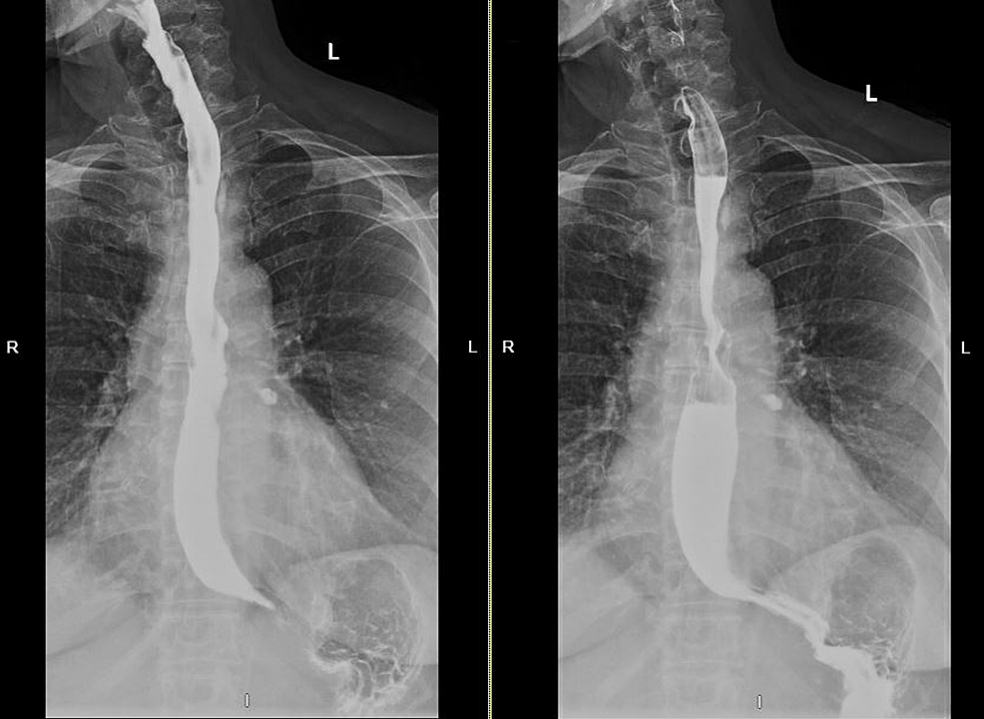
Barium swallow demonstrating narrowing at the gastroesophageal junction with mild gastroesophageal reflux.

**Figure 3:**
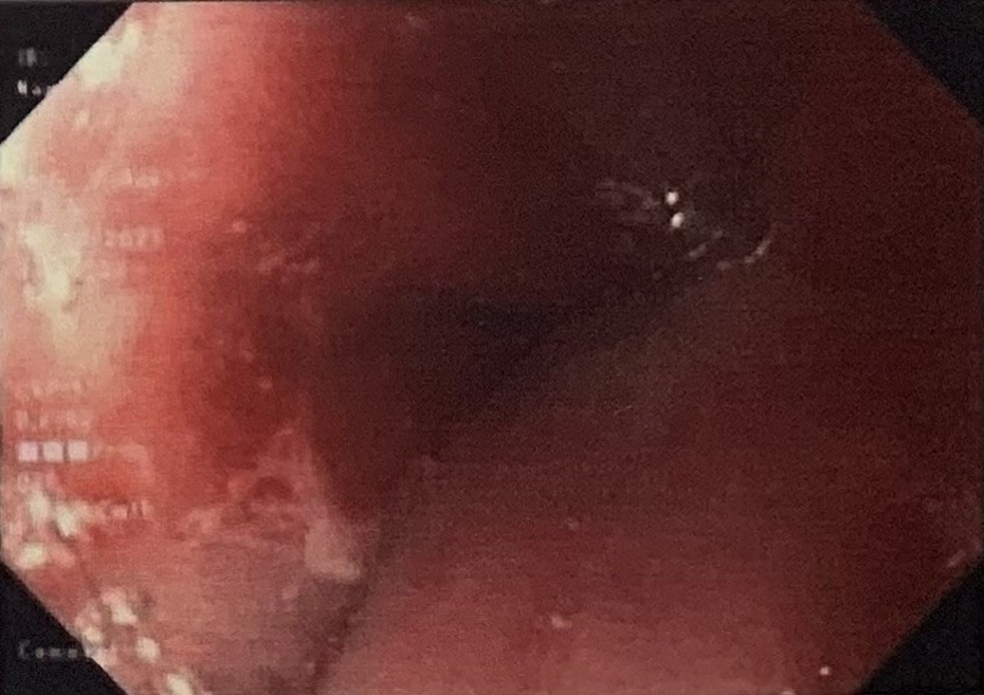
Image from the subsequent endoscopy that was performed to assess both the integrity of the fundoplication and the narrowed segment of the esophagus noted on radiographic imaging. Biopsies from this site revealed heterotopic pancreas on histopathology.

**Figure 4:**
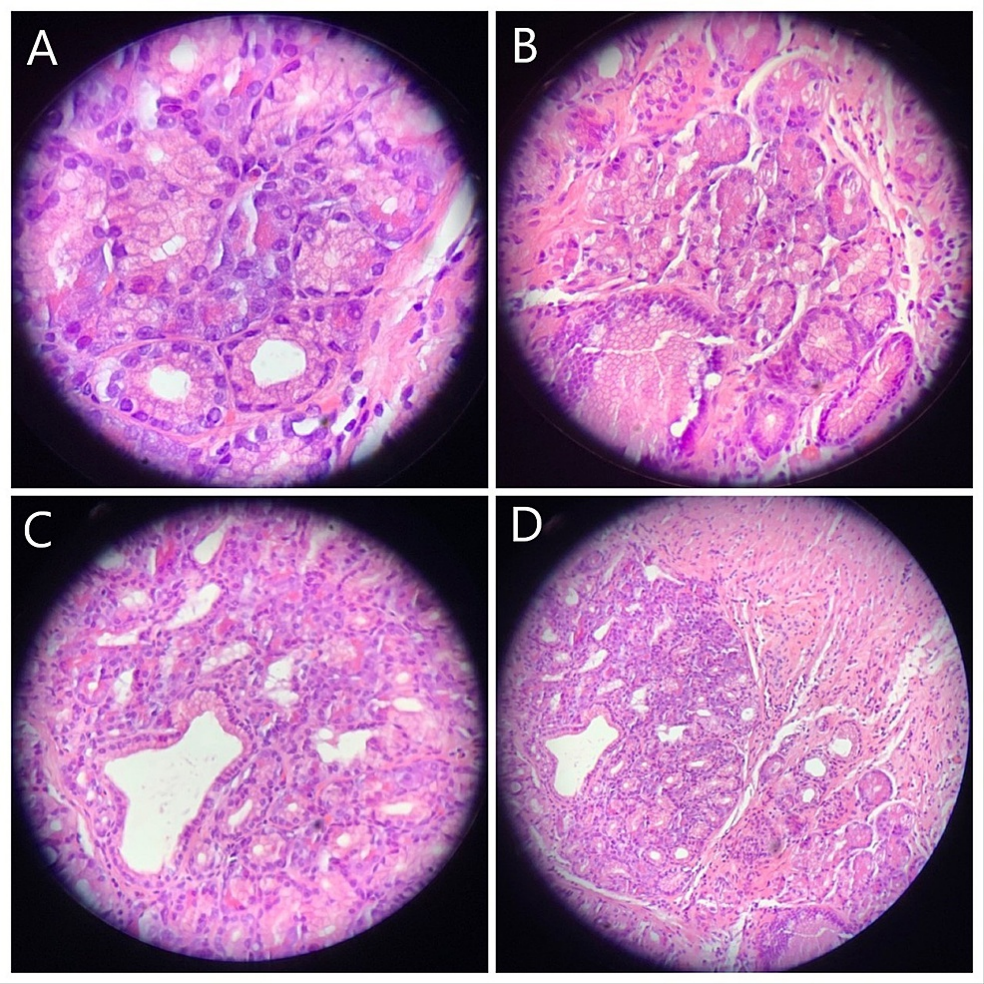
Histologic slides from endoscopic biopsy demonstrating squamoglandular mucosa consistent with gastroesophageal origin surrounding a small focus of ectopic pancreatic tissue.

On follow-up, the patient noted that her symptoms had persisted despite maximum medical therapy. Considering the intestinal metaplasia and focal atypia at the patient’s GEJ, history of large hiatal hernia, and extra-esophageal symptoms, the patient was referred to general surgery for laparoscopic hiatal hernia repair with fundoplication to prevent further reflux and damage to the esophagus. The patient suffered significant nausea prior to the procedure; therefore, preoperative motility studies were unable to be obtained. Due to the inability to obtain results from the motility studies, the decision was made to undergo Toupet fundoplication instead of Nissen fundoplication. On follow-up with the surgeon six weeks later, the patient reported two episodes of reflux symptoms with occasional hoarseness. The patient also noted a nodule at the anterior neck. Subsequent physical examination and thyroid ultrasound were unremarkable, while a barium swallow demonstrated narrowing at the GEJ with mild gastroesophageal reflux (Figure 2). Subsequent EGD was performed to assess both the integrity of the fundoplication and the narrowed segment of the esophagus noted on radiographic imaging (Figure 3). Biopsies from this endoscopy revealed squamoglandular mucosa consistent with GEJ origin and a small focus of pancreatic rest (Figures 4A-4D). Additional histologic examination confirmed no high-grade dysplasia or malignant involvement. The patient is currently undergoing surveillance measures and continuing maximum medical therapy.

## Discussion

Heterotopic pancreas located at the GEJ has been scarcely reported with only five cases existing in the English medical literature. The frequency of heterotopic pancreas on autopsy was between 0.55% and 13.7%, with a male predominance of 3:1 [6]. It has been reported in various portions of the gastrointestinal tract, with 90% of patients having ectopic tissue in either the stomach (38%), duodenum (36%), or proximal jejunum (16%) [7]. The ectopic tissue has also been reported to occur in other less common areas, such as Meckel’s diverticulum, the ileum, common bile duct, umbilicus, splenic hilum [7], gallbladder [8], the fallopian tube [9], and the esophagus [2,10-13].

This is an exceptional case of heterotopic pancreas primarily due to its anatomical location within a hiatal hernia with persistent reflux symptoms after surgical repair. To our knowledge, a pancreatic rest at this location has only occurred in two other reported cases in the literature. Guillou et al. describe a case of the ectopic pancreas at the GEJ within a hiatal hernia of a 60-year-old man. This patient was discovered to have adenocarcinoma at the GEJ during upper endoscopy, which was indicated by worsening dysphagia and weight loss. The tumor was resected along with the proximal part of the stomach through a lower left-sided thoracotomy. On microscopic examination, the mass was consistent with ductal adenocarcinoma originating from the ectopic pancreas. The patient ultimately died of the disease three months postoperatively [11]. Crighton and Botha describe another case of the heterotopic pancreas at the GEJ in a hiatal hernia of a 58-year-old woman. This patient presented with symptoms of worsening dysphagia. She underwent two upper endoscopies, which identified the mass at the GEJ, but biopsies did not reveal the etiology of the mass. She underwent positron emission tomography (PET) after computed tomography (CT) showed some patchy changes in the right lower and right upper lobes of the lungs. The PET showed increased activity in the paraesophageal mass and some mediastinal lymph nodes. A third EGD was performed with endoscopic ultrasound (EUS), which demonstrated an extraluminal, nodular, friable mass in the left hemicircumference. The mass was surgically resected via laparoscopic Ivor Lewis esophagectomy. Histopathology revealed an intraductal papillary mucinous neoplasm (IPMN) arising from a pancreatic rest in the esophageal wall [10].

Salim et al. describe a case with striking similarities to this case with mild differences. They document a 29-year-old male with a past medical history of GERD who presented to the emergency department with epigastric and retrosternal chest pain worsened during fasting. This patient also failed medical therapy with sucralfate and was scheduled for outpatient endoscopy. On follow-up, the patient had worsening dysphagia with solid foods. A barium esophagram demonstrated slight delayed emptying and manometry demonstrated frequent failed swallows and weak peristalsis suggestive of ineffective esophageal motility. No masses or lesions were observed on endoscopy, but an irregularity in the Z-line in the distal esophagus was biopsied, which showed focal pancreatic heterotopia on histology [12].

Shalaby et al. reported a case of a 52-year-old male presenting with chronic episodic dysphagia of solid foods. An endoscopic evaluation revealed a one-centimeter smooth submucosal mass at the GEJ, which was eventually biopsied using EUS. Histologic examination of the biopsied site demonstrated a single gland with partial goblet cell formation that did not reach the surface with heterotopic pancreatic tissue. This patient chose not to undergo surgical resection. Instead, he chose to modify his eating habits by consuming smaller food boluses and chewing his food more thoroughly to prevent dysphagia with solid, larger boluses [13].

The management of heterotopic pancreas at the GEJ differs throughout the literature depending on the size, symptomatology, and potential for malignancy. When the ectopic tissue is located at the GEJ, the patient can present as asymptomatic or they can present with dysphagia due to mass effect, worsening reflux or heartburn, or epigastric pain. Management in these cases ranges from observation with conservative medical therapy, resection, or esophagectomy. The patient in our case presented with worsening reflux symptoms with a hiatal hernia. EUS may have been helpful in the evaluation of this patient, as this modality was commonly utilized in other cases; however, access to this resource was limited in this location of rural Appalachia. After a hiatal hernia repair with Toupet fundoplication, we will continue to maximize conservative medical therapy and monitor the ectopic tissue with surveillance endoscopies and biopsies. If the tissue becomes dysplastic in the future, excision will be necessary.

## Conclusions

Here we report the third case of the heterotopic pancreas, or pancreatic rest, located at the GEJ in a female patient, confirmed via histopathology after endoscopic biopsy. This condition is rare with only about 18 adult cases reported in the English medical literature; seven of which were in women, and five of which originated at the GEJ. Of these five cases, only two occurred in a hiatal hernia. Our patient presented with worsening reflux symptoms and received a hiatal hernia repair with Toupet fundoplication prior to the discovery of the pancreatic rest. After persistent symptoms of reflux, the GEJ was biopsied endoscopically and histologic examination revealed pancreatic rest with no malignant involvement. The patient will be followed with surveillance endoscopies and will continue maximum medical therapy.
